# Hsp20 Protects against Oxygen-Glucose Deprivation/Reperfusion-Induced Golgi Fragmentation and Apoptosis through Fas/FasL Pathway

**DOI:** 10.1155/2015/606934

**Published:** 2015-06-24

**Authors:** Bingwu Zhong, Zhiping Hu, Jieqiong Tan, Tonglin Lu, Qiang Lei, Chunli Chen, Liuwang Zeng

**Affiliations:** ^1^Department of Neurology, Second Xiangya Hospital, Central South University, Changsha, Hunan 410011, China; ^2^Department of Traditional Chinese Medicine, Second Xiangya Hospital, Central South University, Changsha, Hunan 410011, China; ^3^National Key Laboratory of Medical Genetics, Central South University, Changsha, Hunan 410078, China

## Abstract

Cerebral ischemia-reperfusion injury plays an important role in the development of tissue injury after acute ischemic stroke. Finding effective neuroprotective agents has become a priority in the treatment of ischemic stroke. The Golgi apparatus (GA) is a pivotal organelle and its protection is an attractive target in the treatment of cerebral ischemia-reperfusion injury. Protective effects of Hsp20, a potential cytoprotective agent due to its chaperone-like activity and involvement in regulation of many vital processes, on GA were assessed in an ischemia-reperfusion injury model. Mouse neuroblastoma Neuro2a (N2a) cells were subjected to oxygen-glucose deprivation/reperfusion (OGDR) insult. OGDR induces Golgi fragmentation, apoptosis, and p115 cleavage in N2a cells. However, transfection with Hsp20 significantly attenuates OGDR-induced Golgi fragmentation and apoptosis. Hsp20 interacts with Bax, decreases FasL and Bax expression, and inhibits caspases 3 and p115 cleavage in N2a cells exposed to OGDR. Our data demonstrate that increased Hsp20 expression protects against OGDR-induced Golgi fragmentation and apoptosis, likely through interaction with Bax and subsequent amelioration of the OGDR-induced elevation in p115 cleavage via the Fas/FasL signaling pathway. This neuroprotective potential of Hsp20 against OGDR insult and the underlying mechanism will pave the way for its potential clinical application for cerebral ischemia-reperfusion related disorders.

## 1. Introduction

The Golgi apparatus (GA) is a pivotal organelle in cell metabolism and participates in many vital processes, such as oxidative stress, signal transduction, and cell apoptosis. It is supposed to be cellular headquarters where cargo sorting/processing, basic metabolism, signaling, and cell-fate decisional processes converge [1, 2]. Fragmentation of the Golgi is induced from a broad range of insults, such as increased neuronal activity, neurodegenerative disorders, oxygen-glucose deprivation (OGD), and ischemia, indicating that GA is an attractive therapeutic target in many disorders whose etiology is based upon dysfunction of GA [3–6].

p115 is a 961 kDa Golgi-associated peripheral membrane protein. It forms homodimers and mediates several docking steps and vesicle transport. p115 plays a very important role in maintaining the structural organization of the GA, while a cleavage fragment of p115 by caspase results in GA fragmentation and also induces apoptosis [7]. It is suggested that c-terminal fragment of p115 promotes apoptosis through facilitating ERK phosphorylation of p53 [8].

Small heat shock proteins are molecular chaperones and some of them have been suggested to be protective in neurological disorders induced by protein misfolding [9, 10]. Hsp20 is one of the small heat shock proteins. It plays a vital role in modulating many physiological conditions, as well as in protecting against an amount of pathological processes [11]. We previously have demonstrated that Hsp20 attenuates mitochondrial fragmentation and exerts protection against oxygen-glucose deprivation/reperfusion (OGDR) insult [12, 13]. However, the neuroprotective effects of Hsp20 and its underlying mechanisms are still far from clear.

Therefore, on the basis of previous findings, the aims of this study were to determine (1) whether OGDR induced Golgi fragmentation and p115 cleavage in Neuro2a (N2a) cells, (2) whether Hsp20 exerts neuroprotective effects on GA damage induced by OGDR in N2a cells, and (3) the cytoprotective mechanism of Hsp20 after OGDR exposure. We found that OGDR induced Golgi fragmentation and p115 cleavage in N2a cells. Hsp20 attenuated OGDR-induced damage to GA and apoptosis, which is related to interacting with Bax and subsequently ameliorating the OGDR-induced elevation in cleavage of p115 through Fas/FasL signaling pathway. Our results suggest that Hsp20 represents a novel therapeutic option for treatment of a myriad of disorders related to cerebral ischemia-reperfusion injury.

## 2. Materials and Methods

### 2.1. Mouse N2a Neuroblastoma Cells Culture

Mouse N2a neuroblastoma cells were purchased from American Type Culture Collection (ATCC). N2a neuroblastoma cells were used and maintained in Dulbecco's modified Eagle's medium (DMEM), supplemented with 10% FBS (Gibco BRL), 100 U/mL penicillin, and 100 *μ*g/mL streptomycin, at 37°C in a moist atmosphere containing 5% CO_2_.

### 2.2. Plasmid Construction and Transfection

Total RNA was isolated from N2a cell cultures by using of TriZol (Invitrogen). Reverse transcription was performed by using of the Reverse Transcription Kit (Promega). Hsp20 was obtained by using primers with Restriction enzyme* EcoR *I and* BamH* I in 5′-Terminal. The following primer sets were used to generate specific fragments as before [14]: Hsp20 forward, 5′-GCGAATTCATGGAGATCCCCGTGCCTGTGCA-3′, Hsp20 reverse, 5′-GCGGATCCGCCTTGGCAGCAGGTGGTGACGGA-3′. The PCR product was cloned to pEGFPN1 vector using* EcoR *I and* BamH* I. Insert was confirmed by sequencing. Transfections were carried out by using Lipofectamine 2000 as described by the manufacturer. N2a cells were subjected to oxygen-glucose deprivation and reperfusion (OGDR) insult 36 h after transfection.

### 2.3. Oxygen-Glucose Deprivation and Reperfusion (OGDR)

To mimic ischemic-like conditions in vitro, cell cultures were exposed to oxygen-glucose deprivation (OGD) for 4 hours and then returned to 95% air, 5% CO_2_, and glucose-containing medium for different recovery time as before [15]. First, mouse N2a neuroblastoma cells were transferred into a temperature controlled (37°C) anaerobic chamber (Forma Scientific) containing a gas mixture composed of 5% CO_2_, 95%N_2_. The culture medium was replaced with deoxygenated glucose-free Hanks' Balanced Salt Solution (Invitrogen) and cells were maintained in the hypoxic chamber for 4 hours. After OGD, N2a cells were maintained in DMEM supplemented with 10% FBS under normoxic culture conditions for 0, 4, and 12 hours.

### 2.4. Immunofluorescence Staining

Mouse N2a neuroblastoma cells grown on coverslips, transfected with a green fluorescent protein (GFP) or Hsp20-GFP for 36 h, or without transfection, were subjected to OGD for 4 hours followed by reperfusion for 0, 4, and 12 hours. The cells were then fixed with 4% paraformaldehyde for 30 min and washed three times with PBS, pH 7. 4. The cells were incubated with a primary rabbit anti-Golgi58 antibody (1 : 200; Santa Cruz Biotechnology) overnight at 4°C. On the following day, the cells were incubated with fluorescein-conjugated anti-rabbit IgG (1 : 400; Vector Laboratories) for 1 h. N2a cells were counterstained with 1 *μ*g/mL 4′,6-diamidino-2-phenylindole (DAPI) (Vector Laboratories, Burlingame, CA, USA) to visualize nuclear morphology. Slides were washed, wet mounted, and examined with an Olympus confocal fluorescence microscope. Fluorescence pictures were taken with identical exposure settings.

### 2.5. Measurement of Apoptosis

Mouse N2a neuroblastoma cells transfected with a green fluorescent protein (GFP) or Hsp20-GFP for 36 h, were subjected to OGD for 4 hours followed by reperfusion for 12 hours. Apoptosis was detected by Annexin V-FITC Apoptosis Detection Kit (Sigma). Briefly, N2a cells were collected and washed twice with PBS. 500 *μ*L Binding Buffer suspension was then added to the treated cells. After that, 5 *μ*L Annexin V-FITC and 10 *μ*L propidium iodide were added to each group and cultures were incubated at 37°C for 5~15 min in dark. Flow cytometer (BD Biosciences) was used to detect the percent cells with apoptosis and flowJo software was used for flow cytometry analysis.

### 2.6. Western Blot Analysis

Total protein was isolated from the N2a cells using 2XSDS sample buffer (63 mM Tris-HCl, 10% Glycerol, and 2% SDS). Samples (20–40 *μ*g of protein) were electrophoresed onto a 10–15% SDS/polyacrylamide gel (SDS/PAGE) and transferred to PVDF membranes. The membranes were blocked in TBS-Tween buffer containing 20 mM Tris-HCl, 5% nonfat milk, 150 mM NaCl, and 0. 05% Tween-20 (pH 7. 5) for 1 hour at room temperature. Thereafter, the blot was incubated with primary rabbit anti-p115 antibody (ab184014, 1 : 1000; Abcam), FasL (#4273, 1 : 1000, Cell signaling), Bax (ab77566, mouse monoclonal antibody, Abcam), caspase 3 (#14220, 1 : 1000, Cell signaling, 1 : 1000), Hsp20 for IP (ab184161, rabbit monoclonal antibody, 1 : 1000, Abcam), Hsp20 for WB (ab88362, mouse monoclonal antibody, 1 : 1000, Abcam), and mouse anti-actin antibody (1 : 10,000; Santa Cruz) for 1-2 hours at room temperature. The membrane was washed with TBST 3 times at 10-minute intervals, incubated with the secondary antibody (1 : 5000; anti-rabbit or anti-mouse IgG conjugated with horseradish peroxidase; Jackson ImmunoResearch Laboratories) at room temperature for 1 hour, and then washed 3 times each at 10-minute interval with TBST and 2 times each for 10 minutes with TBS. Band was visualized via an enhanced chemiluminescence kit (ECL) according to the manufacturer's suggested protocol (GE Health). Membranes were then exposed to X-ray film.

### 2.7. Coimmunoprecipitation and Western Blot

Rabbit monoclonal and mouse monoclonal anti-Hsp20 antibodies were purchased from Abcam. Rabbit IgG was used for each immunoprecipitation as a control. Monoclonal antibodies to Bax and Hsp20 were used in the Western blots. For the Co-IP assay, the protein concentration in supernatants was adjusted to 1 mg/mL and 0. 5 mL of the lysate was incubated with 2 *μ*g of Hsp20 antibody or rabbit IgG overnight and then incubated with 40 *μ*L of resuspended volume of Protein A/G-agarose beads (BD0045, Bioworld Technology, Inc.) at 4°C for 4 h. Washing was performed four times in the original lysis buffer. Coimmunoprecipitated Hsp20 or Bax was detected by Western blot with Hsp20 or Bax mouse monoclonal antibodies.

### 2.8. Quantitative and Statistical Analysis

For quantitation, fragmented Golgi was defined as scattered dots (not connected) in the perinuclear region or multiple mini-Golgi (isolated dots) dissociated from the major GA [16]. Quantification was performed using more than 300 cells per experiment. Quantitative analysis of selected bands in Western blots was performed by using the NIH Image Analysis System (ImageJ version 1.48). Quantitative data were expressed as mean ± SEM based on at least 3 separate experiments of triplicate samples. Differences among groups were statistically analyzed by one-way analysis of variance followed by Bonferroni's post hoc test. Comparison between two experimental groups was based on a two-tailed *t*-test. Differences between the mean values were considered significant if *P* < 0.05.

## 3. Results

### 3.1. OGDR Induces Golgi Fragmentation and Apoptosis in N2a Cells

**Figure 1:**
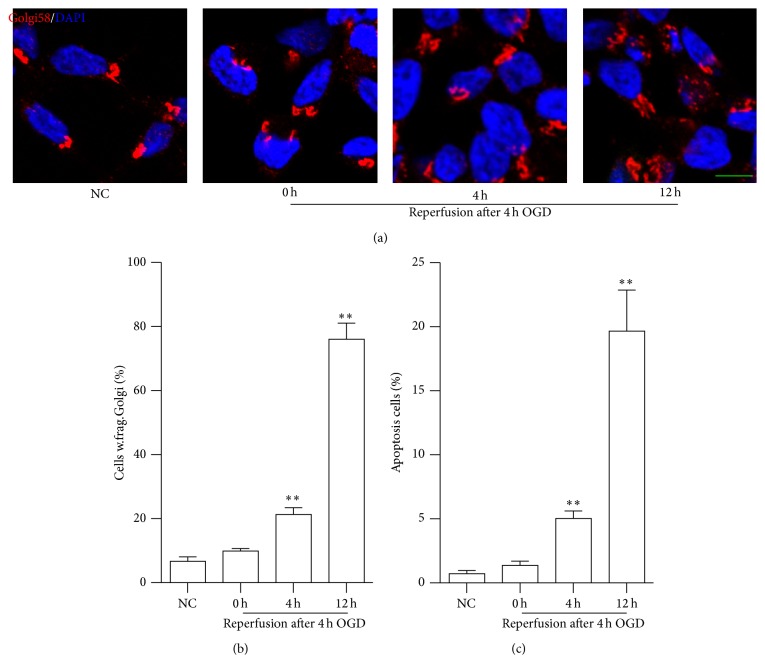
Fragmentation of GA and apoptosis in N2a cells without transfection after OGDR. The experiment was repeated independently for at least three times. (a) Immunofluorescent stain using an antibody against Golgi marker Golgi58 (red color) and counterstain with 4′,6′-diamidino-2-phenylindole (blue color) to show nuclei revealed normal GA in normal conditions and after 4 h of OGD treatment. However, more and more N2a cells showed fragmented GA after 4 and 12 h reperfusion following 4 h OGD. (b) Quantitation (mean ± SEM) of (a) from three independent experiments. The increase of N2a cells with fragmented GA begun as early as 4 h reperfusion following 4 h OGD exposure and further enhances after 12 h reperfusion. (c) Apoptosis of N2a cells after OGDR insult. No significant difference was found between control and 0 h reperfusion following 4 h OGD. However, the proportion of apoptotic cells was significantly increased after 4 and 12 h reperfusion. ^*∗∗*^ *P* < 0.01 compared to normal conditions (Bar = 10 *μ*m).

To explore whether Golgi fragmentation occurs in N2a cells without transfection upon OGDR insult, we used immunofluorescent staining to evaluate its temporal profiles (Figure 1). The increase of Golgi fragmentation in a time-dependent manner was found during the different time points of OGDR. As demonstrated in Figure 1(a), most of GA appeared to be ribbon-like structures adjacent to the nuclei in normal conditions. After 4 h of OGD treatment, most of GA still appeared to be ribbon-like structures adjacent to the nuclei. However, after 4 and 12 h reperfusion following 4 h of OGD, the morphology of GA changed to debris-like structures scattered in the cytoplasm. The increase of N2a cells with fragmented GA began as early as 4 h reperfusion following 4 h OGD exposure and further enhanced after 12 h reperfusion (Figure 1(b)).

By using flow cytometry, Figure 1(c) depicts time-dependent cellular apoptosis of N2a cell after OGDR treatment. There was no significant difference of apoptotic cells after 0 h reperfusion following 4 h OGD compared with control. However, after 4 and 12 h reperfusion, the proportion of apoptotic cells was significantly increased compared with control.

### 3.2. OGDR Induces p115 Cleavage in N2a Cells

**Figure 2:**
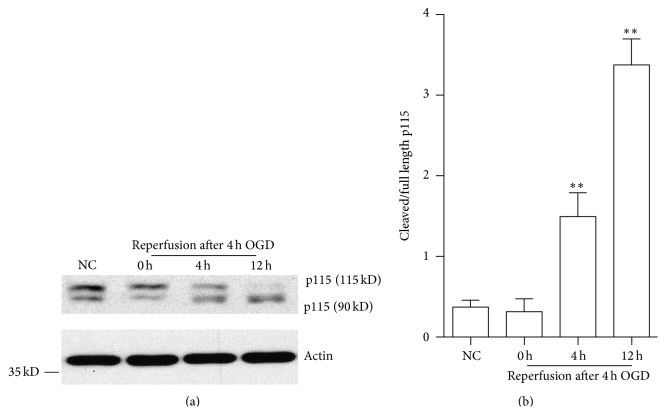
P115 cleavage in N2a cells after OGDR. The experiment was repeated independently for at least three times. (a) The increase of p115 cleavage after OGDR exposure. (b) Quantitation (mean ± SEM) of (a) from three independent experiments. The OGDR-induced injury was accompanied by an increase in p115 cleavage, which begun as early as 4 h reperfusion following 4 h OGD and further enhanced after 12 h reperfusion. ^*∗∗*^ *P* < 0.01 compared to normal conditions.

GA undergoes irreversible fragmentation during the process of apoptosis. GA fragmentation partly results from cleavage of golgins, several high molecular weight coiled-coil proteins including the Golgi vesicle tethering protein p115, mediated by caspase. Proteolysis of the C-terminus of the Golgi protein p115 contributes significantly to Golgi fragmentation and has been shown to promote apoptosis [17]. The increase of p115 cleavage in a time-dependent manner was found during the different time points of OGDR. As demonstrated in Figure 2, p115 cleavage to the 90 kDa fragment occurred more efficiently after OGDR insult than in control cells. The increase of p115 cleavage begun as early as 4 h reperfusion following 4 h OGD exposure and further enhanced after 12 h reperfusion. Therefore, the OGD 4 h plus 12 h reperfusion model was used for further experiments.

### 3.3. Hsp20 Protects against OGDR-Induced Golgi Fragmentation and Apoptosis

**Figure 3:**
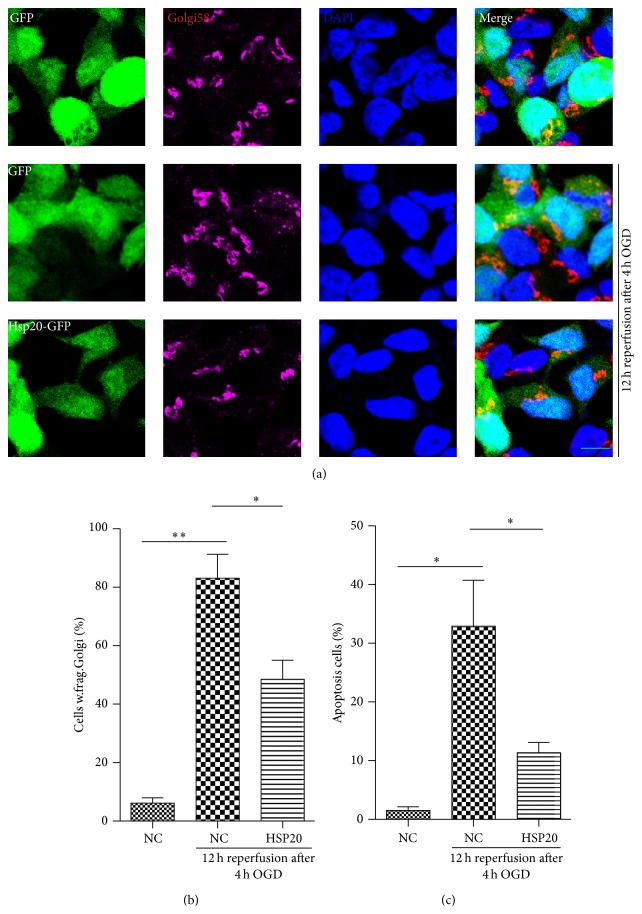
Effects of Hsp20 on GA morphology and apoptosis in N2a cells exposed to OGDR. N2a cells were transfected with different plasmids (pEGFP-N1 and pEGFP-Hsp20). After transfection for 36 h, cells were treated with OGD 4 h plus 12 h reperfusion. The experiment was repeated independently for at least three times. (a) Digital photomicrograph under fluorescent illumination showing the morphology of GA was detected using Golgi58 staining. GA displayed typical ribbon-like structures adjacent to the nuclei in N2a cell transfected with pEGFP-N1 without OGDR exposure. Fragmented GA was evident in N2a cells transfected with pEGFP-N1 exposed to 4 h OGD plus 12 h reperfusion insult. Transfection with Hsp20 significantly attenuated OGDR-induced fragmentation of GA. (b) Quantitation (mean ± SEM) of (a) from three independent experiments. (c) Overexpression of Hsp20 in vitro reduced OGDR-induced apoptosis. Values are expressed as mean ± SEM. ^*∗*^ *P* < 0.05 compared to control. ^*∗∗*^ *P* < 0.01 compared to control (Bar = 10 *μ*m).

To further analyze the effects of Hsp20 on OGDR-induced Golgi fragmentation, N2a cells were transfected with different plasmids (pEGFP-N1 and pEGFP-Hsp20). After transfection for 36 h, cells were treated with OGD 4 h plus 12 h reperfusion. Results showed that, in N2a cell transfected with empty vector (pEGFP-N1) without OGDR exposure, GA displayed typical ribbon-like structures adjacent to the nuclei (Figure 3(a)). When N2a cell transfected with pEGFP-N1 were exposed to OGD 4 h plus 12 h reperfusion, lots of GA changed to debris-like structures scattered in the cytoplasm. Meanwhile, transfection with Hsp20 significantly attenuated OGDR-induced Golgi fragmentation (Figures 3(a) and 3(b)).

To determine whether Hsp20 affected the induction/or efficiency of apoptosis, after transfection with pEGFP-N1 and pEGFP-Hsp20 plasmids for 36 h, N2a cells were subjected to OGD 4 h plus 12 h reperfusion (Figure 3(c)). The percentage of apoptotic cells was increased after OGD 4 h plus 12 h reperfusion. However, N2a cells transfected with pEGFP-Hsp20 displayed a significant decrease in the number of apoptotic cells after OGDR exposure. Together, these data strongly suggest that increased Hsp20 expression ameliorates OGDR-induced Golgi fragmentation and apoptosis.

### 3.4. Hsp20 Interacts with Bax

**Figure 4:**
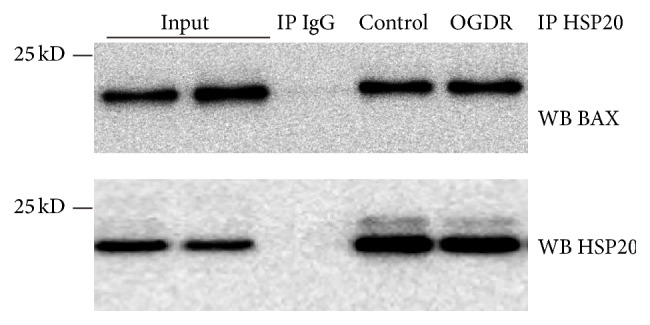
Hsp20 interacts with Bax. Co-IP of Bax with Hsp20. The co-IP was performed with anti-Hsp20 antibodies and an IgG control, followed by Western blot with anti-Bax antibodies. The experiment was repeated independently for at least three times.

In our previous results, Hsp20 was shown to protect against mitochondrial fragmentation, regulate Bcl-2 and Bax expression, and reduce the release of cytochrome c from mitochondria to cytosol upon OGDR insult [12, 13]. To further clarify the cytoprotective mechanism of Hsp20, coimmunoprecipitation (co-IP) assay was used to examine whether Hsp20 may interact with Bax. As shown in Figure 4, we found that anti-Hsp20 antibodies, but not the IgG control, specifically coimmunoprecipitated Bax, suggesting that Bax interacted with Hsp20 in N2a cells, which is in consistency with the results in human hepatocellular carcinoma cells [18].

### 3.5. Hsp20 Decreases FasL and Bax Expression and Inhibits Caspases 3 and p115 Cleavage in N2a Cells Exposed to OGDR

**Figure 5:**
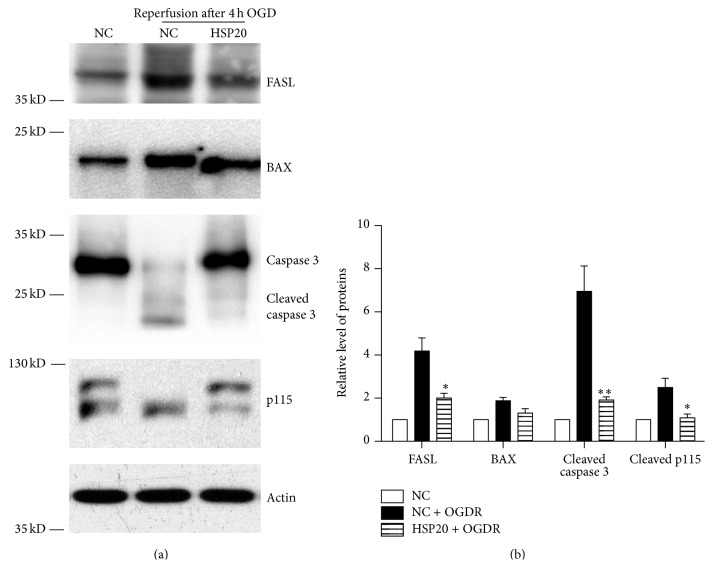
Hsp20 decreases FasL and Bax expression and inhibits caspases 3 and p115 cleavage in N2a cells exposed to OGDR. The experiment was repeated independently for at least three times. (a) Effects of Hsp20 on apoptotic proteins expression and p115 cleavage in N2a Cells treated with OGD 4 h plus 12 h reperfusion. Transfection with Hsp20 decreased FasL and Bax expression and inhibited caspases 3 and p115 cleavage after 12 h reperfusion following 4 h OGD. (b) Quantitation (mean ± SEM) of (a) from three independent experiments. Values are expressed as mean ± SEM. ^*∗*^ *P* < 0.05 compared to N2a Cells treated with OGD 4 h plus 12 h reperfusion. ^*∗∗*^ *P* < 0.01 compared to N2a Cells treated with OGD 4 h plus 12 h reperfusion.

To determine whether Hsp20 modulated the expression of apoptotic proteins and p115 cleavage, N2a cells were transfected with pEGFP-N1 and pEGFP-Hsp20 and treated with OGD 4 h plus 12 h reperfusion (Figure 5). Western blotting results revealed that FasL expression, caspases 3, and p115 cleavage were significantly increased in N2a Cells transfected with pEGFP-N1 after OGD 4 h plus 12 h reperfusion. However, the enhancement of FasL expression, caspases 3, and p115 cleavage was significantly inhibited in N2a Cells transfected with Hsp20. The increased expression of Bax was also inhibited in N2a cells transfected with Hsp20, though not significantly. Together, these data strongly suggest that increased Hsp20 expression ameliorates OGDR-induced elevation of apoptotic proteins and p115 cleavage.

## 4. Discussion

Acute ischemic stroke is one of the leading causes of death and a first frequent cause of permanent disability in adults worldwide. Though great advances have been achieved in the understanding of its physiology and pathophysiology, treatment option for acute ischemic stroke is still limited [19]. Not only does a reduced cerebral blood supply lead to substantial brain tissue damage, but also reperfusion contributes to the poor outcomes after ischemic stroke. Cerebral ischemia and subsequent reperfusion lead to cellular organelles damage and triggers Golgi dispersal. In the study, the ribbon-like GA changed to debris-like structures scattered in the cytoplasm in N2a cells subjected to 4 and 12 h reperfusion following 4 h of OGD, which mimic ischemia/reperfusion-like conditions in vitro. Meanwhile, apoptosis of N2a cells also increased after OGDR insult.

Significantly, Golgi fragmentation has been observed in patients with neurodegenerative diseases, such as Alzheimer's disease (AD) [16, 20], amyotrophic lateral sclerosis (ALS) [21, 22], spinocerebellar ataxia [23], corticobasal degeneration, and Creutzfeldt-Jakob disease [24]. Fragmentation of GA is thought to be a common characteristic of neurodegenerative diseases. Meanwhile, Golgi fragmentation occurred in OGD-induced cerebral vascular endothelial cells death [5] and in the cortical cells of gerbils after ischemia-reperfusion injury [6]. We demonstrated that OGDR induced Golgi fragmentation, suggesting that dispersal of Golgi is also a common feature of cerebral ischemia-reperfusion injury.

As we know, GA is a pivotal organelle in cell metabolism, functioning not only in the processing and transportation of cargoes but also in ion homeostasis, cell apoptosis, and stress sensing. Therefore, Golgi fragmentation and dysfunction in N2a cells after OGDR insult may affect not only trafficking, modification, and processing of protein and its processing enzymes, but also of many other proteins critical for cellular functions and cause global changes on the cell surface [16, 21, 25]. Furthermore, fragmentation of neuronal GA is supposed to be an early and likely irreversible change during the process of neurodegeneration that induces apoptosis. Golgi fragmentation and subsequent collapse after OGDR insult are an essential process in the development of cell death [26]. As a result, our study suggests that protection of GA is an attractive option in the treatment of cerebral ischemia-reperfusion injury.

As suggested above, dispersal of Golgi is a common characteristic of cerebral ischemia-reperfusion injury and many neurodegenerative disorders. However, the molecular mechanism underlying the fragmentation of the Golgi is far from clear. It is demonstrated that the fragmentation of GA occurring in apoptosis is partly due to cleavage of its structural proteins by caspase, such as the Golgi-vesicle-tethering protein p115 [8]. P115 is a 962-residue peripheral membrane protein present in the intermediate compartment and cis-Golgi vesicles that functions in endoplasmic reticulum to Golgi trafficking. P115 associates with gamma-tubulin and plays a critical role in Golgi structure and mitosis progression. It is the only known golgin to regulate both mitosis and apoptosis [27]. Short interfering RNA-mediated knockdown of p115 [28] or cleavage of p115 [7, 17, 29] induced extensive Golgi fragmentation and apoptosis. In the study, we found that p115 cleavage to the 90 kDa fragment was increased after OGDR insult. Thus, the enhanced p115 cleavage would contribute to extensive OGDR exposure induced Golgi fragmentation. Meanwhile, cleavage of p115 also induces apoptosis [7, 17, 29]. The enhanced cleavage of the C-terminus of the Golgi protein p115 after OGDR exposure would also contribute to promoting N2a cells apoptosis. As a result, inhibition p115 cleavage is a potential therapeutic target for neuroprotection after cerebral ischemia-reperfusion injury.

Hsp20 (HspB6) is constitutively expressed in various tissues and transiently upregulated in response to cellular stress/damage [30, 31]. Hsp20 possesses chaperone-like activity and “housekeeping” roles and is involved in regulation of many vital processes [11, 32, 33]. In the nervous system, Hsp20 is supposed to be a potential neuroprotective agent and plays a central role in the neuroinflammatory reaction and contributes to cognitive decline in Alzheimer's disease (AD) [34]. OGDR insult also regulates the expression of Hsp20 and Hsp20 attenuates mitochondrial fragmentation after OGDR treatment [12, 13]. In this study, we found that enhanced Hsp20 expression significantly attenuated OGDR-induced Golgi fragmentation and apoptosis. As suggested above, GA is a pivotal organelle in neurons and is a potential drug target for cerebral ischemia-reperfusion injury treatment. Therefore, Hsp20 may exert neuroprotection against OGDR by maintaining GA morphology and inhibition of apoptosis, indicating that modulating Hsp20 expression could be beneficial in the treatment of cerebral ischemia-reperfusion injury.

We further investigated the cytoprotective mechanism of Hsp20 upon OGDR insult. In previous studies, we have demonstrated that Hsp20 could protect against OGDR-induced mitochondrial fragmentation, regulate Bcl-2 and Bax expression, and reduce the release of cytochrome c from mitochondria to cytosol [12, 13]. Based on these results, in this study, we identified an interaction between Hsp20 and Bax by co-IP assay, suggesting that Hsp20 is a Bax-interacting protein. Therefore, our results raise the interesting possibility that Hsp20 may inhibit OGDR-induced Golgi fragmentation and apoptosis by preventing the activation of Bax.

Furthermore, the multiple functions attributed to Hsp20 suggest the intriguing possibility that the Bax-Hsp20 complex may function in multiple signalling pathways. The Fas (CD95)/Fas ligand (CD95L) system plays a vital role in regulating apoptosis. Binding of Fas by its ligand FasL would result in activation of caspase 8 and subsequently activate downstream caspases, leading to several critical regulatory proteins cleavage, such as p115 [35]. The Fas/FasL system was upregulated in myocytes during hypoxia, ischemia, and ischemia-reperfusion [36]. Our results demonstrated that OGDR insult enhanced the expression of Fas-L and Bax, as well as cleavage of caspases 3 and p115, suggesting that apoptosis via the Fas/Fas ligand signalling system plays an important role in the development of ischemia-reperfusion injury. However, overexpression of Hsp20 significantly inhibited the activation of Fas/FasL and its downstream signaling pathway, leading to decreased cleavage of caspases 3 and subsequently p115. As suggested above, cleavage of p115 contributes to Golgi fragmentation and induces apoptosis [7, 8]. Therefore, Hsp20 protects OGDR-induced Golgi fragmentation and apoptosis by inhibition of p115 cleavage. Taken together, these studies demonstrate that Hsp20 plays an important role in maintaining survival of N2a cells after OGDR insult, which is mediated by Fas/FasL signaling pathway and subsequently decreased cleavage of p115.

**Figure 6:**
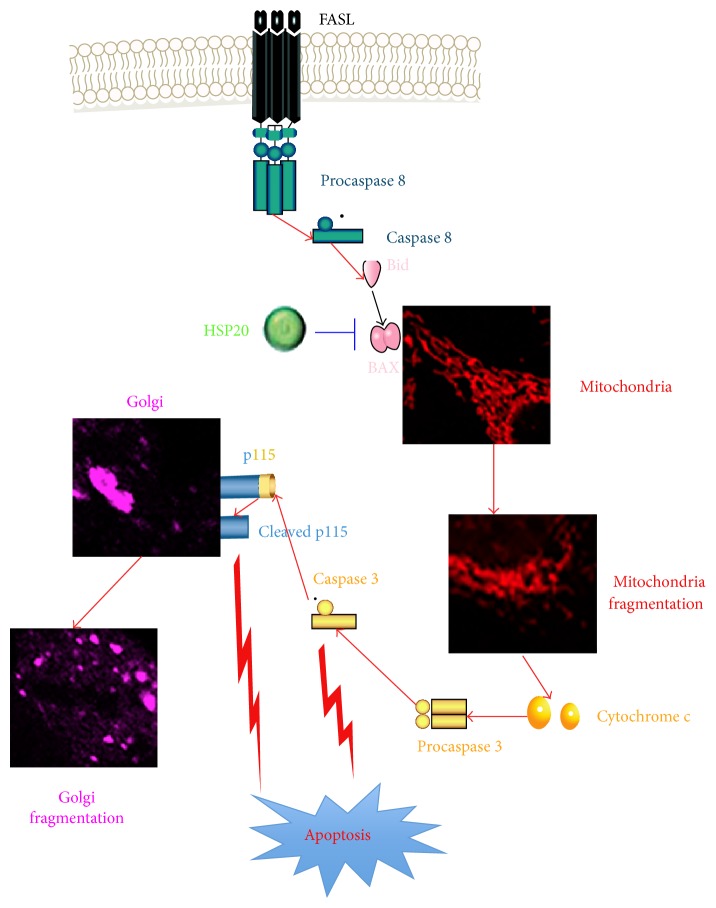
The potential neuroprotective mechanism of Hsp20 upon OGDR insult.

In conclusion, our study demonstrates that OGDR disrupts GA and induces apoptosis and p115 cleavage in mouse N2a neuroblastoma cells. Hsp20 protects against OGDR-induced Golgi fragmentation and apoptosis, indicating its cytoprotective role in cerebral ischemia-reperfusion injury. The neuroprotective mechanism may be related to interacting with Bax and subsequently ameliorating the OGDR-induced elevation in cleavage of p115, which is mediated by Fas/FasL signaling pathway (Figure 6). Our results showed the beneficial effects of Hsp20 against OGDR, which would pave the way for its potential clinical application. Further efforts may lead to the development of novel therapies for disorders whose etiology is based upon cerebral ischemia-reperfusion injury by using of Hsp20.
